# Indigenous Infection with *Francisella tularensis holarctica* in The Netherlands

**DOI:** 10.1155/2013/916985

**Published:** 2013-09-02

**Authors:** Boulos Maraha, Gerhard Hajer, Andreas Sjödin, Mats Forsman, Armand Paauw, Guus Roeselers, Ellen Verspui, Ine Frenay, Daan Notermans, Maaike de Vries, Frans Reubsaet

**Affiliations:** ^1^Department of Medical Microbiology, Beatrix Hospital and Albert Schweitzer Hospital, P.O. Box 899, 3300 AW Dordrecht, The Netherlands; ^2^Department of Surgery, Beatrix Hospital, P.O. Box 90, 4200 AB Gorinchem, The Netherlands; ^3^Division for CBRN Defence and Security, FOI—Swedish Defence Research Agency, Umeå, Sweden; ^4^Department of Earth, Environmental, and Life Sciences, TNO, P.O. Box 45, 2280 AA Rijswijk, The Netherlands; ^5^Public Health Service Zuid-Holland Zuid, P.O. Box 166, 3300 AD Dordrecht, The Netherlands; ^6^Diagnostic Laboratory for Infectious Diseases and Perinatal Screening (LIS), Center for Infectious Disease Control, National Institute of Public Health and the Environment (RIVM), P.O. Box 1, 3720 BA Bilthoven, The Netherlands

## Abstract

We report here the first case of indigenous tularemia detected in The Netherlands, a nonendemic country, since 1953. Whole genome DNA sequence analysis assigned the isolate BD11-00177 to the genomic group B.FTNF002-00, which previously has been exclusively reported from Spain, France, Italy, Switzerland, and Germany. The patient had not been abroad for years, which implies that this is an indigenous infection. The current case might predict an upcoming distribution of *Francisella tularensis holarctica* genomic group B.FTNF002-00 in Europe.

## 1. Introduction

Tularemia is a zoonotic infection caused by *Francisella tularensis*, a small Gram-negative coccobacillus. Transmission to humans has been reported by direct contact with infected animals, arthropod bite, inhalation of contaminated dust, and ingestion of contaminated food or water. The clinical presentation depends on the mode of transmission and the strain involved. In Europe and Asia, only *F. tularensis* subspecies *holarctica* (type B) is present, whereas in North America, also the more virulent *F. tularensis* subspecies *tularensis* (type A) is found. *F. tularensis* subspecies *mediasiatica* is restricted to central Asia. Human infections with *F. novicida*, *F. hispaniensis* and *F. philomiragia* are exceedingly rare [1–4]. In addition to the clinically relevant species, the *Francisella* genus also contains several other species not infecting humans [5, 6]. *F. tularensis* is a reemerging pathogen, and there have been recently increasingly reports on tularemia in Europe [7]. We report here the first case of indigenous tularemia detected in The Netherlands, a non-endemic country, since 1953.

## 2. Case Presentation

A previously healthy 72-year-old Dutch male presented in October 2011 with fever (39.3°C), a vesicular lesion on the forehead, and periauricular lymphadenopathy on the right side. A common bacterial skin infection was suspected, and amoxicillin-clavulanic acid was administrated. However, a week later, the patient developed a preauricular swelling of 5 by 2.5 cm. An ultrasound of the swelling showed an inhomogeneous preauricular swelling, originating from the parotid gland. Pus was collected from the swelling for culture. In the Gram stain, no microorganisms were seen. Auramine staining and PCR for mycobacteria were negative. Culture yielded growth of thin Gram-negative rods. The identification of the isolate with the routine tests was inconclusive. Subsequently, the National Institute of Public Health and the Environment (RIVM) identified the isolate as *F. tularensis* subspecies *holarctica* using an in-house PCR on the *tul4* (*lpnA*) gene and an in-house PCR on the helicase gene [8]. The patient was treated with intravenous ciprofloxacin (400 mg twice daily) and gentamicin (4 mg/kg once daily) for two weeks. He had a rapid clinical response to the therapy, and the swelling had diminished. After two months, he was fully recovered. No evidence of relapse was seen at a 12-month followup. 

The patient is a florist, regularly visiting an auction to buy flowers, imported from all over the world. He did not recall having been bitten by any insect during his work. No colleagues had been ill during the same period. He had not been abroad for years, and he had no contact with diseased animals. However, the patient was bitten by insects during a boat trip in a wetland area in the north of The Netherlands a week before he became ill. The wetland area is approximately 45 km from the German border, a country where increasing numbers of tularemia have been reported [9].

## 3. Discussion 

Tularemia is an endemic disease in many European countries [4, 9–11]. However, the only previous known case of indigenous tularemia in The Netherlands was documented in 1953, when seven family members were infected after eating a hare infected with *F. tularensis* subspecies *holarctica* [12]. The current case suggests that tularemia is distributed in Europe, even in non-endemic countries. Factors contributing to an increase in the incidence of the disease may include arthropod proliferation, enlargement of animal reservoir and increased contact with the reservoir of *F. tularensis* [7]. The rarity of tularemia in non-endemic countries contributes to difficulties in early diagnosis of the disease. Our patient presented with ulceroglandular tularemia, which usually follows a bite of an infected arthropod. The patient had two possible risk exposures: an arthropod bite during the boat trip and an unnoticed arthropod bite during his flower business. As no other cases of tularemia have been noticed recently, the source of infection in our case remains uncertain. 

**Figure 1:**
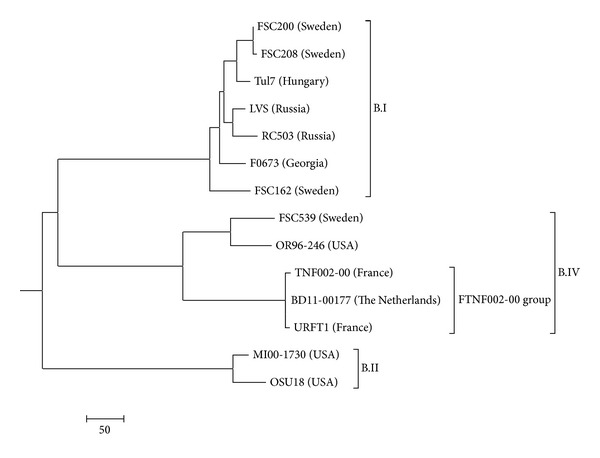
Evolutionary relationships of *Francisella tularensis* subspecies *holarctica* strain BD11-00177. The new isolate, BD11-00177, from The Netherlands belongs to the FTNF002-00 genomic group.

It has been mentioned by other investigators that genetic and phylogenetic analyses of *F. tularensis* subspecies *holarctica* isolates in Europe contribute to better understanding of the movement of this pathogen between European countries [10]. To further investigate the possible source of the infection in the current case, the isolate, denoted BD11-00177, was sequenced using Roche 454 and Illumina MiSeq technology. The sequence reads from the isolate are deposited at NCBI Bioproject under accession number PRJNA177784. The assembled draft genome sequence was compared with publicly available *F. tularensis* genome sequences. This analysis showed that the isolate belongs to the Franco-Iberian subclone of *F. tularensis* subsp. *holarctica* strains firstly described by Dempsey et al. [13] and subsequently denoted FTNF002-00 genomic group (B.Br.FTNF002-00 and BIV.FTNF002-00) defined by the FTNF002-00 genome sequence [4, 14, 15]. The presence of the 1.59 kb RD23 deletion event [13] as well as the 464 bp size of the MLVA marker FtM24 [16], both typical for the FTNF002-00 genomic group, was confirmed in silico. Previously, isolates from this genomic group have exclusively been reported from Spain, France, Italy, Switzerland, and Southwest Germany [4, 10, 13, 16–18]. Evolutionary history of *F. tularensis* subspecies *holarctica* strain BD11-00177 was inferred using the neighbor-joining method [19]. The tree in Figure 1 is drawn to scale, with branch lengths in the same units as those of the evolutionary distances used to infer the phylogenetic tree. The evolutionary distances were computed using the number of differences method and are in the units of the number of base differences per sequence [20]. The analysis involved 14 *F. tularensis* subspecies *holarctica* genome sequences using *F. tularensis* subspecies *tularensis* strain SCHU S4 as outgroup. All positions containing gaps and missing data were eliminated. There was a total of 1599589 positions in the final dataset. Evolutionary analyses were conducted in MEGA5 software [21]. 

In conclusion, we report for the first time the detection of FTNF002-00 genomic group of *F. tularensis* subspecies *holarctica* in The Netherlands. *F. tularensis* subspecies *holarctica* might have been recently introduced in The Netherlands following the emergence in other European countries. Therefore strengthened surveillance is required. Tularemia should be considered in the differential diagnosis in patients presenting with lymphadenopathy and cutaneous lesion even in non-endemic countries.
